# Autoantibodies against C1q as a Diagnostic Measure of Lupus Nephritis: Systematic Review and Meta-analysis

**DOI:** 10.4172/2155-9899.1000210

**Published:** 2014-04-22

**Authors:** Paul Eggleton, Obioha C. Ukoumunne, Isabel Cottrell, Asma Khan, Sidra Maqsood, Jemma Thornes, Elizabeth Perry, David Isenberg

**Affiliations:** 1 Institute of Biomedical and Clinical Sciences, University of Exeter Medical School, University of Exeter, Exeter, UK; 2 NIHR CLAHRC South West Peninsula (PenCLAHRC),University of Exeter Medical School, University of Exeter, Exeter, UK; 3 Centre for Rheumatology, Department of Medicine University College London, UK

**Keywords:** Autoantibody, Biopsy, Diagnosis, Enzyme-linked immunosorbent assay (ELISA), Hierarchical summary receiver operating characteristic (HSROC), First component of complement (C1q), Systemic lupus erythematosus (SLE)

## Abstract

**Objectives:**

To evaluate the diagnostic accuracy of C1q autoantibodies in identifying lupus nephritis (LN) in patients with systemic lupus erythematosus (SLE).

**Data sources and methods:**

Citation indexes were searched and 370 articles published from 1977 to 2013 were evaluated. The 31 selected studies included in the meta-analysis were cross-sectional in design. Among the 31 studies, 28 compared anti-C1q antibodies in 2769 SLE patients with (n=1442) and without a history of LN (n=1327). Nine studies examined anti-C1q in 517 SLE patients with active (n=249) and inactive LN (n=268). Hierarchical summary receiver operating characteristic (HSROC) random effects models were fitted to pool estimates of accuracy across the studies.

**Results:**

Anti-C1q antibodies discriminated between patients with and without a history of LN, with a median specificity of 73.5%. The HSROC model estimated the corresponding sensitivity to be 70.4%. A hypothetical patient with a 55% prior probability of having a history of LN as opposed to no history (the median prevalence across 28 eligible studies) would have a post-test probability of 76.4% following a positive test result (positive predictive value) or 33.0% following a negative test result (negative predictive value). For discriminating active from inactive LN the median specificity of anti-C1q antibodies was 80%, with a corresponding estimated sensitivity value 75.7% based on the HSROC model. A hypothetical patient with a 56% prior probability of active as opposed to inactive LN (the median prevalence across the 9 eligible studies) would have a post-test probability of 82.8% following a positive test result or 27.9% following a negative test result.

**Conclusions:**

Although C1q antibodies are associated with lupus nephritis the post-test probabilities are not sufficiently convincing to provide reasonable certainty of the presence or absence of history of disease/active disease.

## Introduction

**Figure 1:**
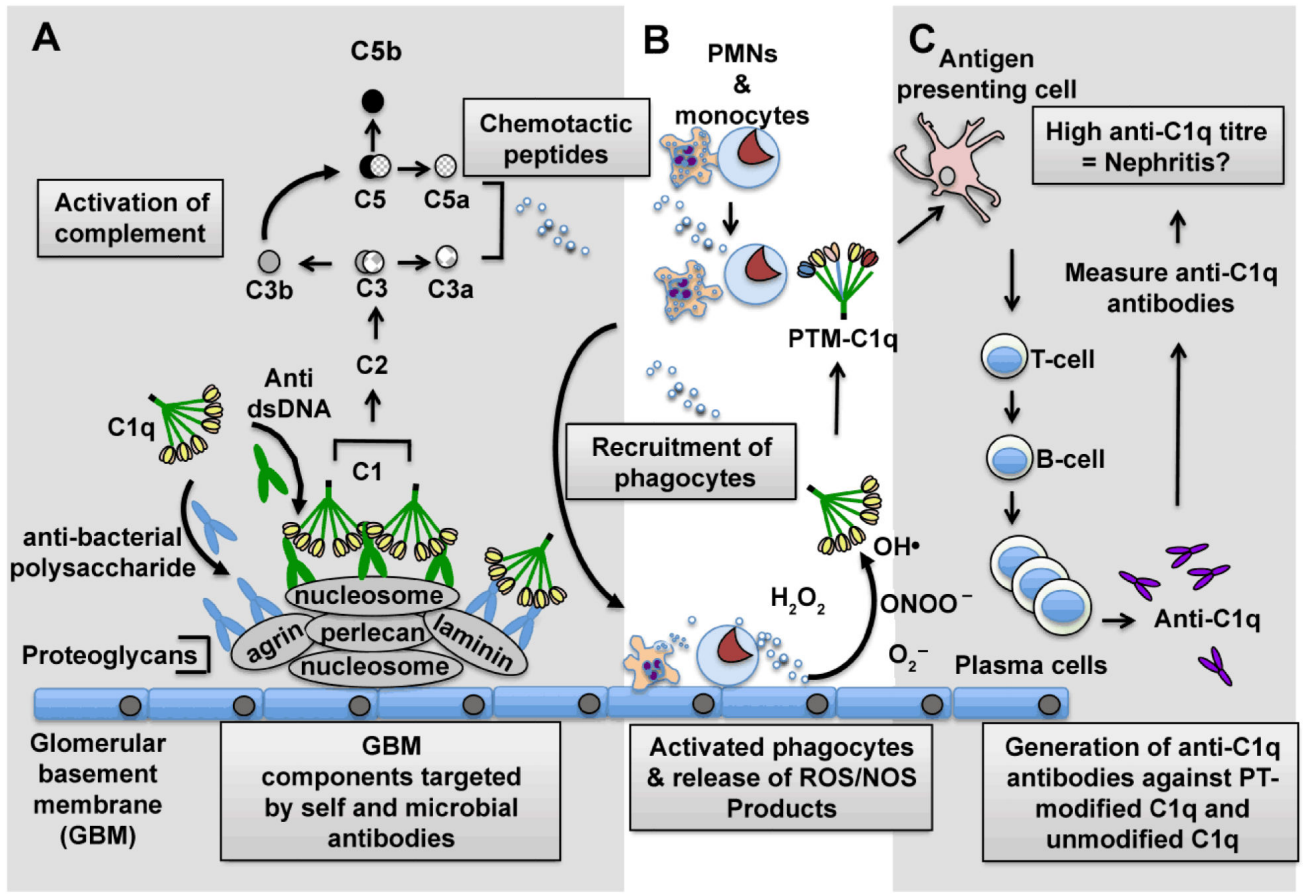
Postulated sequence of events in the generation of anti-C1q antibodies that may act as diagnostic biomarkers of glomerulonephritis. Nucleosome blebs from apoptotic cells can deposit on the glomerular basement membrane (GBM) in SLE patients with LN and associate with a number of proteoglycan molecules. (A) During infection and/or inflammation anti-bacterial polysaccharide or anti-dsDNA antibodies bind to host proteoglycans and nucleosomes, respectively. This leads to the deposition of C1 (C1q/C1r_2_/C1s_2_) on the GBM and subsequent complement activation. (B) The release of chemotactic peptides C5a and C3a triggers the recruitment of phagocytes to the GBM in close proximity to C1q. The activation of the phagocytes leads to the release of free radicals that can post-translationally modify (PTM) C1q. (C) The PTM-C1q can be taken up by antigen presenting cells, and the modified peptides presented to T-cells. The autoreactive T-cells in turn can trigger B-cell activation and ultimately the production of anti-C1q-producing plasma cells. The concentration of anti-C1q antibodies produced in the blood can then detected by various immunoassays, including ELISA and used to assess whether a patient has or has not got nephritis.

The first component of complement – C1 is comprised of three subcomponents, C1q, C1s and C1r. The C1 complex plays a pivotal role in the activation of the classical pathway of complement. Classical complement activation has both inflammatory and anti-inflammatory functions. Intensive research in the 1970s afforded detailed information on the structure and function of C1q [1]. The C1q molecule is a 460 kDa glycoprotein with an exquisite tulip-like structure, consisting of six globular heads each made up from three polypeptide chains – A, B and C. Each head is attached to a central fibril region by a triple helical collagen like tail. The C1q component of C1 is synthesized in monocyte/macrophages and once secreted, can bind to aggregated antibody [2] primarily on microorganisms. This event triggers the activation of the classical complement pathway that in turn amplifies the innate and adaptive immune responses against infectious agents. C1q is a multi-functional protein [3], and binds to immune complexes deposited on tissues, including the kidney [4], and aids in their solubilization and removal [5]. C1q also plays a role in apoptotic cell debris removal [6]. Forty years ago, the possibility of antibodies against C1q in SLE patients was raised [7]. It was later proposed that binding of C1q to immune complexes led to conformational changes in the C1q structure exposing neoepitopes [8] that may invoke an immune response. Evidence for such a response, was demonstrated by Uwatoko et al., who observed that IgG from SLE patient sera cross-reacted with C1q [9]. In later studies, we and others, suggested that post-translational modifications of C1q upon exposure to free radicals could generate antigenic neoepitopes [10-13] which could act as a ‘trigger’, leading to the breakdown of immune tolerance to C1q; this effect together with ‘epitope spreading’ could then provoke the generation of antibodies to both post-translationally modified and unmodified forms of C1q (Figure 1). The binding of anti-C1q antibodies and other proteins to C1q is potentially of concern as it may impede the ability of C1q to carry out its normal anti-inflammatory functions such as, immune complex clearance and removal of apoptotic debris [14,15].

SLE is a multisystem autoimmune disorder with a broad spectrum of clinical presentations. Due to the heterogeneity of the disease and the absence of a single diagnostic test the diagnosis of SLE remains challenging [16]. Current clinical practice requires integration of patient’s symptoms, physical examination and diagnostic tests. Lupus nephritis (LN), a marker of adverse outcome in SLE is common developing in approximately 30-50% of patients overall often in the first year after diagnosis [17]. The cumulative relapse rate for LN is in the region of 25-40% at 5 years [18] with patients experiencing multiple episodes of active nephritis at increased risk of progressing to end stage renal disease [19]. Early recognition of LN is imperative to facilitate treatment however it is clinically and histologically heterogeneous. Diagnosis and monitoring of LN remains a considerable clinical challenge [20,21].

Many autoantibodies are prevalent in unselected SLE patients. Up to 70% of patients have autoantibodies against single stranded-DNA (ssDNA), 40-70% of patients have double-stranded DNA (dsDNA) autoantibodies and around 95% of patients have anti-nuclear (ANA) antibodies [22]. ANA and anti-ssDNA have been useful markers of SLE disease in general, but have low specificity and are found in many other types of musculoskeletal disorders and in infection diseases [23]. In the 1990s, a number of studies proposed that autoantibodies against C1q in SLE patients might be pathogenic and be associated with nephritis severity [24,25]. This led to a series of cross-sectional studies in both Europe and the USA aimed at determining if a correlation existed between the presence and concentration of anti-C1q antibodies in SLE patient sera and the severity of their nephritis [26-32]. In one early study, Siegert et al. concluded that anti-C1q autoantibodies do not correlate with general SLE disease activity, but found a positive correlation between anti-C1q antibody titers and nephritis [30]. Since then numerous other studies have assessed the usefulness of measuring anti-C1q as a non-invasive means of detecting and monitoring LN in SLE patients. The majority of these studies agree that measurement of anti-C1q antibodies is a useful additional serological marker for monitoring LN. However, due to the low frequency of SLE in the general population, many of the single and multicenter studies have recruited relatively small numbers of patients between 15 and 250 individuals. Several studies have concluded anti-C1q is not a useful marker for LN [33], others ‘slightly’ useful [34] and others very useful for predicting renal disease [35]. This has resulted in a lack of confidence in measuring anti-C1q in a clinical setting and to date it is not used routinely as a diagnostic test for LN.

Given the importance of the potential association between anti-C1q antibodies and LN, we systematically reviewed and performed a meta-analysis of the accuracy of anti-C1q amongst SLE patients to distinguish a) between those with and without history of LN, and b) between those with active and inactive LN.

## Studies and Methods

### Identification, selection and quality assessment of studies

**Figure 2:**
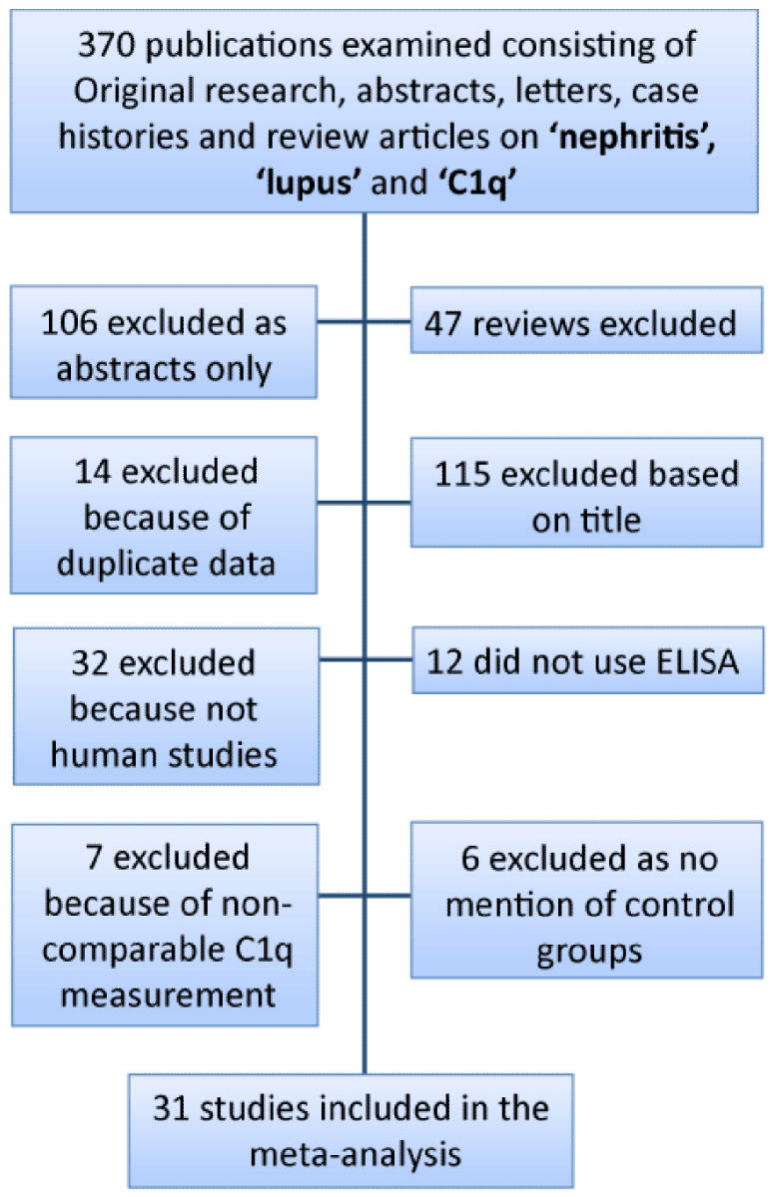
Flow chart for the systematic selection of studies for inclusion in the meta-analysis.

We searched the terms ‘nephritis’, ‘lupus’ and ‘C1q’ in the ‘any field’ bar of EndnoteX4.0.2 using several online search libraries – PubMed (290 citations), Web of Science -TS (197 citations), National Library of Medicine-USA (2 citations) of which 176 references were duplicates (Figure 2). In addition, Annual Reviews, Science Direct, Medline (EBSCO), Biomed Central, BMJ Journals, Cambridge Journals, EBSCO EJS, Oxford Journals, Medline (Ovid), NHS Evidence, AMED (EBSCO) and the Exeter Health Library online journal collection were searched to ensure all relevant articles were retrieved. Four authors (IC, AK, SM and JT) in pairs independently assessed the studies for the accuracy of C1q autoantibodies to diagnose LN as measured by an enzyme-linked immunosorbant assay (ELISA). We also examined the references of all the publications we identified to ensure we had not omitted publications other authors had identified. When data were difficult to extract from the papers, the corresponding authors were contacted and given the opportunity to respond.

Studies were chosen that had employed a lab-made or commercial ELISA to screen for anti-C1q autoantibodies in 12 or more patients per study.

QUADAS-2 (quality assessment of diagnostic accuracy studies) was used to evaluate the risk of bias and applicability of all the studies included in the meta-analysis [36] (http://www.bris.ac.uk/quadas/quadas-2/). The tool assesses the quality of patient selection, the appropriateness of the index test employed (anti-C1q ELISA), the quality of the reference standard as the criterion for disease (lupus nephritis) and the flow and timing of the study (time of sample collection and analysis).

### Statistical analysis

The study-specific results were extracted in the form of 2×2 contingency tables (relating the test result to disease status) for analysis. Sensitivity and specificity estimates are reported for each study using coupled forest plots. Sensitivity/specificity values for the studies are also shown on summary receiver operating characteristic (sROC) plots. Hierarchical summary receiver operating characteristic (HSROC) random effects meta-analysis models [37] were fitted to pool estimates of accuracy of anti-C1q for discriminating between patients with and without history of LN and between patients with active LN and with inactive LN. The HSROC model recognizes the statistical heterogeneity across studies and the trade-off between sensitivity and specificity that results from the use of different thresholds to define a positive result on the anti-C1q test. The fitted model defines a summary ROC curve with a specific position (accuracy) and shape. Here we report the sensitivity value on the fitted summary ROC curve that corresponds to the median of the specificity values across the studies. Positive and negative likelihood ratios are reported and in turn used to calculate post-test probabilities of nephritis following a positive or negative anti-C1q test result for patients with a pre-test probability of disease (or prevalence) equal to the median percentage with nephritis across the studies. The post-test probability of nephritis following a positive test result is the positive predictive value and the post-test probability following a negative test result is the complement of the negative predictive value.

The HSROC models were fitted using a Bayesian framework in WinBUGS software (http://www.mrc-bsu.cam.ac.uk/bugs/winbugs). Rutter and Gatsonis [37] have described the model in detail. Uniform priors were specified for the accuracy, threshold (representing the cut-point on anti-C1q to indicate a positive result) and shape (representing the degree of asymmetry of the summary ROC curve) model parameters and inverse-gamma priors were specified for the parameters that represent between-study variance for accuracy and threshold. Starting values were set to zero for the accuracy and threshold parameters and to 0.2267 for the shape parameter; to 5 for the between-study variance components for accuracy and threshold; and to zero for the study-specific random effects for accuracy and shape. For estimation, a burn-in of 10,000 iterations was used followed by a further 100,000 iterations for the main run to monitor the posterior distributions. Revman 5.2 software was used to produce the couple forest plots and the summary ROC plots [38].

## Results

### Demographic characteristics of the patients

The meta-analysis included 31 studies in total (Table 1). Twenty eight studies with a total of 2769 SLE patients provided data to compare anti-C1q test result between those with (n = 1442) and without (n=1327) a history of nephritis. Nine studies with a total of 517 SLE patients provided data to compare anti-C1q test result between those with active (n=249) and in active LN (n=268) at the time of blood sampling.

The ethnicities of the studies were diverse and included studies from Europe (17/31 studies; 55%), Asia (9 studies; 29%), North and South America (5 studies; 16%). Twenty nine of the studies assessed anti-C1q status and LN in adults and some teenagers (age ≥ 15 years, range 15-77), four studies focused on pediatric patients (mean age 13.9 years). Patients were recruited into the various studies with a diagnosis of SLE ranging from 2 months to 49 years (Table 1). The percentage of participants that was female exceeded 80% in 22 of the 24 studies that reported these data. The majority of the studies diagnosed SLE (30/31) using one of the American College of Rheumatology (ACR) classifications [39,40]. A large proportion of studies (23 of 31) also used a disease activity index to assess renal activity (Table 2), with the SLEDAI and SLEDAI-2K being the most frequently used (21 studies). The studies in which only ACR criteria were used tended to be the earlier studies conducted between 1994 and 2000. Active and inactive LN was assessed using a variety of well-established clinical parameters, including excessive proteinuria, increase in creatinine and/or the presence of red blood cells or cellular casts in the urine. As shown in Table 2, there was no unified consensus how active nephritis was monitored. The majority of the studies (28/31) used a renal biopsy as the reference standard to diagnose active LN or historical evidence of past episodes of nephritis, only 3 studies did not report confirmatory biopsy for evidence of nephritis.

### Detection and measurement of anti-C1q antibodies by ELISA

The studies used in this analysis all utilized at least one or more ELISA-based immunoassays. Both lab-made and commercial anti-C1q ELISA’s are used by various labs worldwide, and the decision to use one type rather than another may be based on economic reasons rather than assay precision (Table 2). Many of studies assessed both the presence or absence of anti-C1q and the titer of C1q antibodies in their disease cohorts. In this current investigation, we selected studies that used ELISA-based methods so that general comparisons could be made between studies.

One compounding observation that arose from this analysis was the differences in the selection of a cut-off value for anti-C1q antibody positivity (Table 2). Each study set its own criterion for ‘lab-made’ assays. Studies employing commercial assays in some instances changed the cut-off values as recommended by the manufacturers.

### Quality assessment

**Figure 3:**
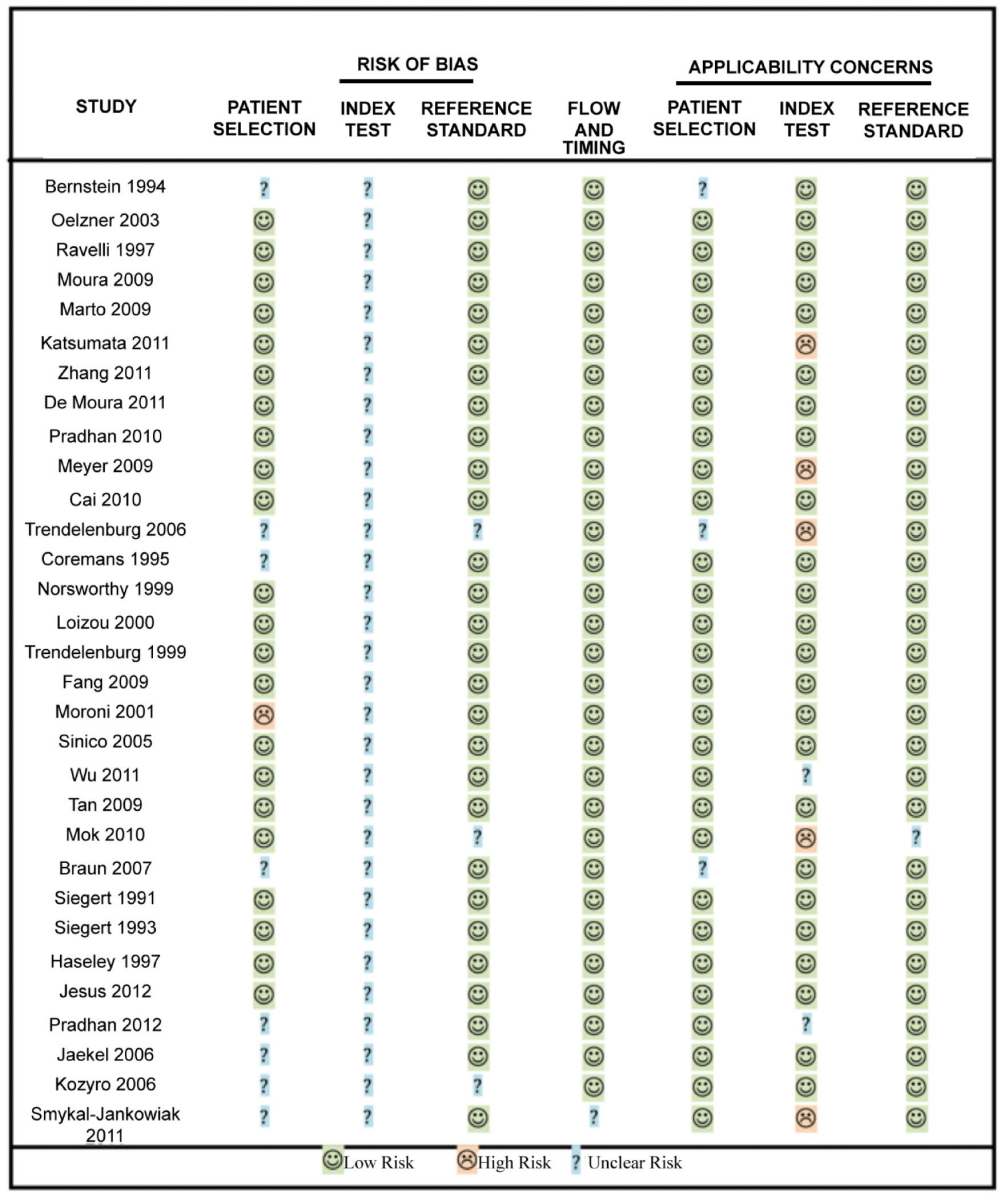
QUADAS-2 quality assessment of selected studies based on inclusion rated in terms of bias and applicability.

The quality of the individual studies is reported in Figure 3 for the different criteria on the QUADAS-2 assessment tool in a format recommended by the QUADAS-2 design team. The majority of studies were cross-sectional in nature, recruiting unselected or consecutive patients into their studies over a number of months or years. Twenty-eight of the 31 studies were retrospective in nature. It was not possible to ascertain from 30 studies whether the anti-C1q assay was performed without any prior knowledge of the nephritis status of the patient samples. In many routine diagnostic studies, evaluations are frequently conducted blind to avoid bias. Only one study claimed to use the anti-C1q ELISA diagnostically. The majority of the studies (28/31) performed a renal biopsy in most of the patients to confirm LN. In 24 studies, proteinuria levels were used as a means to detect nephritis activity. Detailed analysis of raised creatinine levels was performed in 12 studies and the frequency of red blood cells/high powered field of view, was recorded in 16 studies (Table 2). The majority of studies took blood samples at the time of biopsy or disease activity assessment. The resulting isolated sera were routinely batched stored at either −20°C or −80°C prior to being assayed for anti-C1q autoantibodies.

The selected studies scored high for patient selection and use of appropriate clinical assessment of nephritis. However, we identified 7 studies in which the recommended cut-off values distinguishing a positive or negative result were not adhered too; for this reason the studies were graded as having ‘high risk’ concerns. However, an explanation for changing the cut-off values for the anti-C1q tests was provided in the analyzed studies. The main reason given for adjusting the ELISA cut-off value was to meet the needs of the individual studies based on their own non-SLE control subject analysis. In some studies the use of mean OD values ± 1 or more SD were used, with no explanation as to why their results were not presented as ELISA units/ml.

### Diagnostic ability of anti-C1q to distinguish between SLE patients with history of LN and those without a history of LN

**Figure 4:**
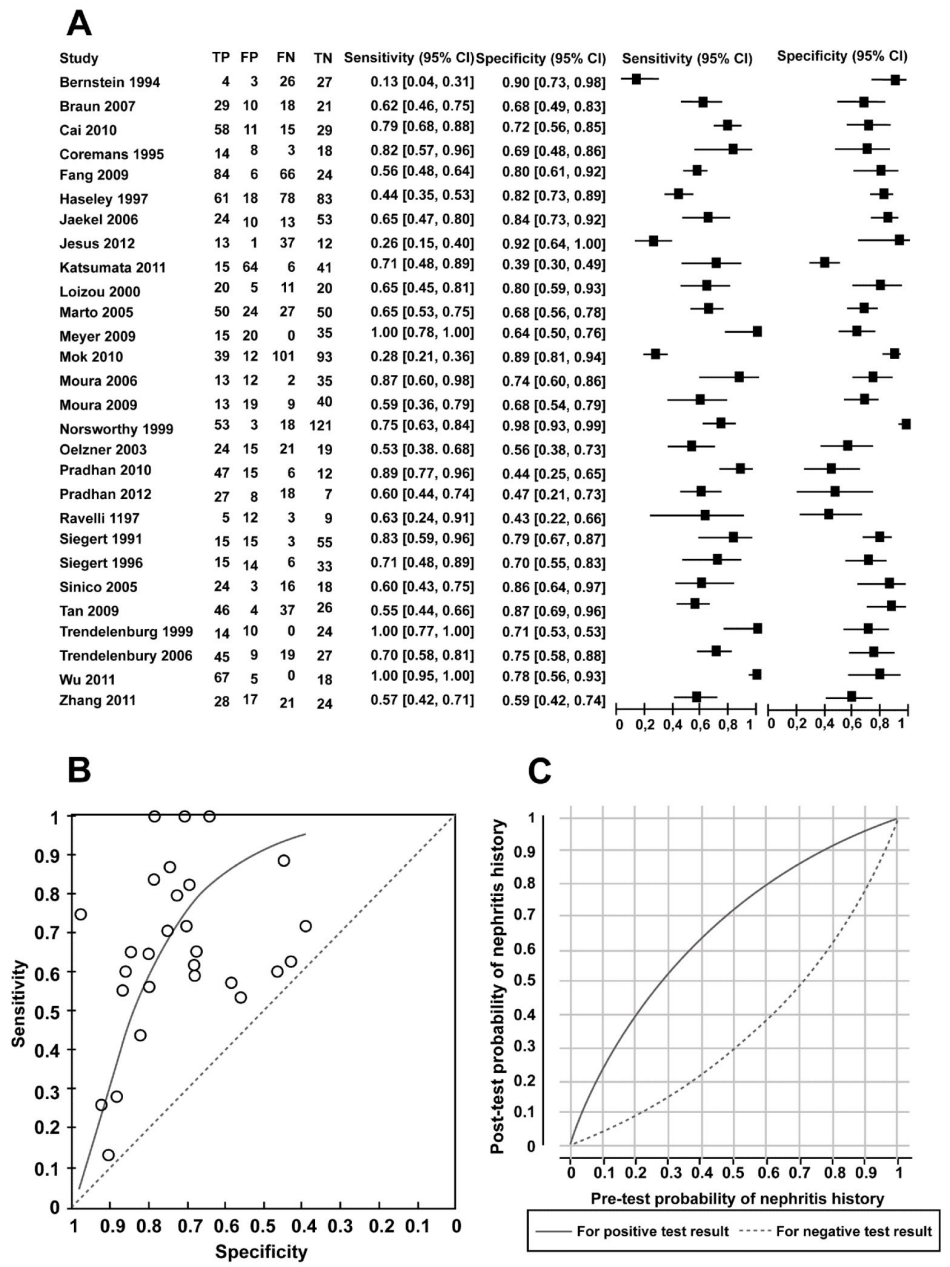
Comparing anti-C1q between patients with and without a history of lupus nephritis. (A) Coupled forest plot of sensitivity and specificity of anti-C1q for distinguishing between patients with and without a history of LN. The sensitivity and specificity values for each individual study are shown (squares) with 95% confidence intervals (horizontal lines). TP – true positives; FP – false positives; FN – false negative; TN – true negatives. (B) Summary ROC plot summarizing sensitivity and specificity of anti-C1q for distinguishing between patients with and without a history of LN. Summary ROC curve based on the fitted HSROC random effects model is shown. Each circle represents an individual study. Points above the diagonal line indicate that the test has better classification than random assignment to a positive or negative test result. (C) Post-test probability of LN history versus pre-test probability. Separate curves shown based on a positive anti-C1q result and a negative anti-C1q result.

Twenty eight studies provided data on the accuracy of anti-C1q to distinguish patients with a current or past history of LN from those with no history of LN. Figure 4A shows the coupled forest plot reporting the sensitivity and specificity estimates from the studies. The sensitivity and specificity points are displayed in ROC space in Figure 4B, with the estimated summary ROC curve from the fitted HSROC model drawn on the plot. The median of the specificity values at study level was 73.5% and the estimated corresponding sensitivity estimated by the HSROC model was 70.4% (95% Credible Interval (CrI): 57.4% to 81.6%). The positive and negative likelihood ratios were 2.66 and 0.40, respectively. If we apply the likelihood ratio values to a population where the underlying proportion of subjects with a history of nephritis is 55% (the median prevalence across the 28 studies) a positive test result would increase the probability of nephritis history to 76.4% and a negative test result would reduce the probability to 33.3%. Figure 4C illustrates the post-test probabilities of a history of nephritis that corresponded to different pre-test probabilities (prevalence values), separately for those with positive and those with negative anti-C1q test results. For a test with high predictive value the curve for positive results would be close to the top of the graph and the curve for negative results close to the bottom. The figure shows that across most underlying prevalence values the anti-C1q test result does not discriminate well and generally leaves uncertainty about the presence of absence of a history of nephritis. In the few scenarios (pre-test probability values above 70%) where the post-test probability of nephritis after a positive anti-C1q result was large enough to be certain that the condition was present the post-test probability after a negative result was not sufficiently low to rule out the condition.

### Diagnostic ability of anti-C1q to distinguish between SLE patients with active LN and those with inactive LN

**Figure 5:**
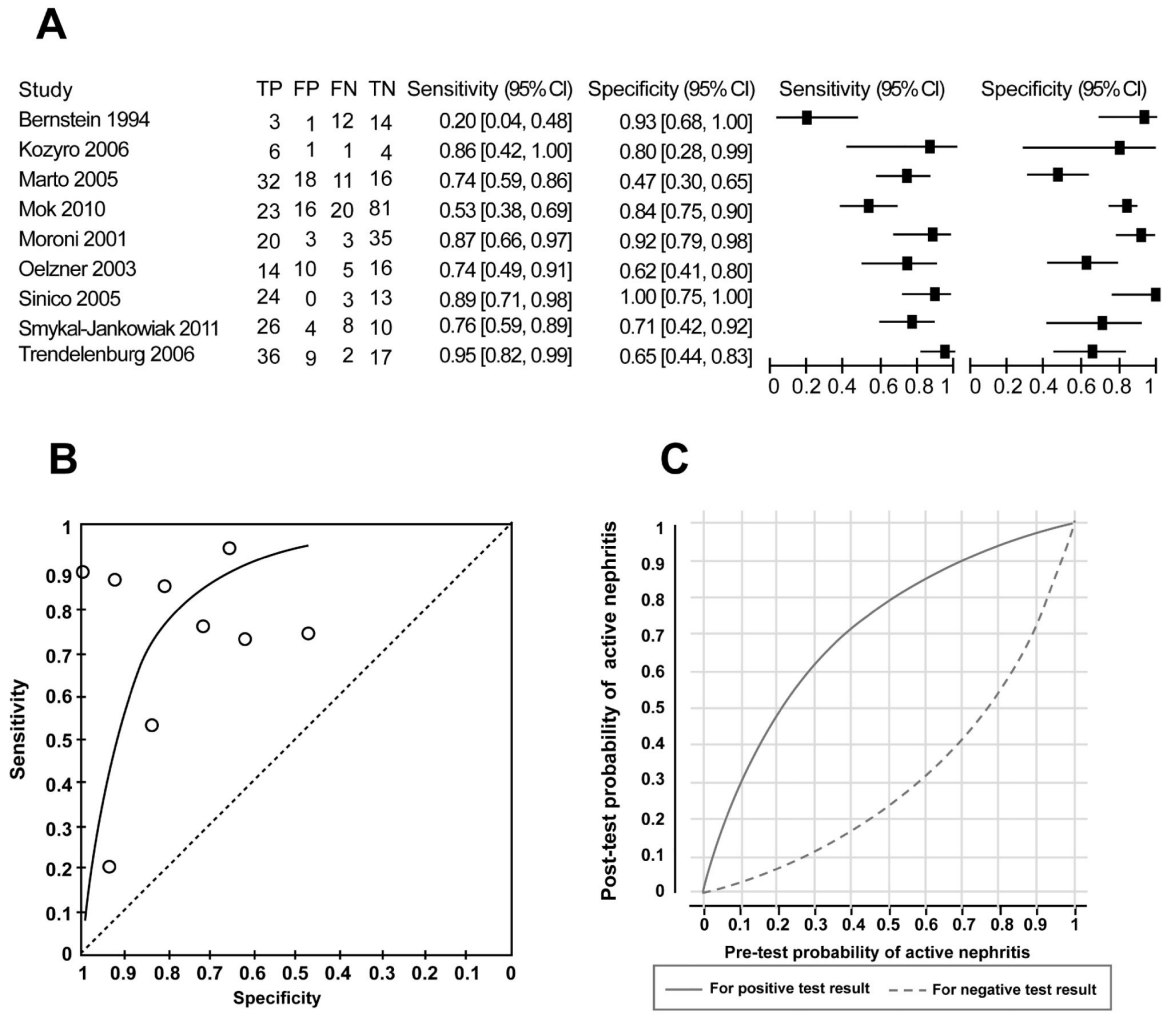
Comparing anti-C1q between patients with active and inactive lupus nephritis. (A) Coupled forest plot of sensitivity and specificity of anti-C1q for distinguishing between patients with active LN and those with inactive LN. The sensitivity and specificity values for each individual study are shown (squares) with 95% confidence intervals (horizontal lines). TP – true positives; FP – false positives; FN – false negative; TN – true negatives. (B) Summary ROC plot summarizing sensitivity and specificity of anti-C1q for distinguishing between patients with active LN and those with inactive LN. Summary ROC curve based on the fitted HSROC random effects model is shown. Each circle represents an individual study. Points above the diagonal line indicate that the test has better classification than random assignment to a positive or negative test result. (C) Post-test probability of active nephritis versus pre-test probability. Separate curves shown based on a positive anti-C1q result and a negative anti-C1q result.

Nine studies provided data on the accuracy of anti-C1q for distinguishing patients with active nephritis from those with inactive nephritis. Figure 5A shows the coupled forest plot with sensitivity and specificity values and Figure 5B shows the study estimates in ROC space with the fitted curve from the HSROC model superimposed. The median of the specificity values of the studies was 80% and the HSROC model estimated sensitivity that corresponds to this was 75.7% (95% CrI: 46.8% to 91.3%). The positive and negative likelihood ratios were 3.79 and 0.30, respectively. Applying these to a population of LN subjects where the probability of active LN is 56% (the median prevalence across the 9 studies), a positive test result would increase the probability to 82.8% and a negative test result would reduce the probability 27.9%. Again, the respective post-test probabilities corresponding to the range of pre-test probability values are generally not sufficiently extreme to ‘rule in’ active LN given a positive test result nor rule it out given a negative test result, as illustrated in Figure 5C.

## Discussion

There is a persisting need for lupus biomarkers that can diagnose active organ involvement during SLE disease flares. Ahearn et al. have recently highlighted this and the difficulties in identifying a specific biomarker to diagnose SLE [41,42]. Among these difficulties, Ahearn highlighted they were required to aid in a) the under- and over-diagnosis of SLE, b) identification of lupus flares, c) stratification of patients with various organ involvements and d) monitoring of therapeutic interventions. To this end over 50 potential biomarkers have been investigated for monitoring SLE [43]. Of these anti-DNA, anti-nucleosome, monocyte chemoattractant protein-1, neutrophil gelatinase-associated lipocalin, urinary tumor necrosis factor (TNF)-like weak inducer of apoptosis, soluble cellular vascular adhesion molecules, C4d levels on erythrocytes, biopsy positive C4d and anti-C1q autoantibodies have all been investigated as renal disease biomarkers. Of these renal biomarkers, anti-C1q has persisted over three decades as a means of monitoring LN in SLE patents in research studies. Autoantibodies against C1q were originally detected against the collagen-like tail region of C1q [29,44], but more recently it has been shown that anti-C1q antibodies are also generated against the A, B and C chains of the globular heads of C1q [45]. Our own studies have suggested oxidative modifications of common host proteins such C1q may lead to breakdown of immune tolerance [46]. C1q has abundant cysteine, methionine and phenylalanines, which are susceptible to attack by reactive oxygen species that can lead to post-translational modifications and possible breakdown of immune tolerance by generating ‘foreign-appearing’ epitopes. Nitrating species such as peroxynitrite can also modify amino acids to form stable end products such as 3-nitrotyrosine that can be immunogenic [47]. This may lead to the generation of unwanted anti-C1q antibodies that can be exploited to monitor disease activity in SLE patients [48].

Many studies performed in the 1990s in northern Europe and the USA assessed anti-C1q as a biomarker for detecting LN in SLE patients. Most were enthusiastic, and whilst European centers continue to assess anti-C1q, those in the USA appear less keen in using anti-C1q to monitor LN. There has been a resurgence of interest in assessing anti-C1q as a biomarker of LN in Asia and South America, particularly in juvenile SLE patients, where renal involvement is a little more frequent than in adults [49]. In our review of 28 studies measuring anti-C1q antibodies to detect a history of LN in SLE patients and 9 studies in which anti-C1q measurement was used to distinguish between active LN and inactive LN, the post-test probabilities after a positive test result were generally too low to be reasonably certain of the presence of the condition. Similarly the post-test probabilities after a negative test result were generally too high to rule out the condition with confidence. These findings apply across most of the range of hypothetical values for the prevalence of nephritis history/active nephritis and suggest the measurement of anti-C1q auto-antibodies as a ‘stand-alone’ biomarker is not diagnostically useful.

The sensitivity and specificity values were highly variable across the included studies (Figures 4 and 5). There are a number of possible reasons for this. Some of these factors were cited in a previous meta-analysis by Yin et al. [50], and included detection methods employed (assay errors) and ethnicity (genetic/environmental) factors. In our analysis we included studies in which commercial and non-commercial ELISA methods were utilized, A frequently used commercial assay is the Bühlmann ELISA. One recent large study of 223 SLE patients monitored anti-C1q antibodies using this and another commercial assay [51]. This individual study was not included in our current meta-analysis, but the corresponding author provided additional sub-cohort data (personal communication – H Julkunen) of their study. The sensitivity, specificity, positive predictive value, and negative predictive value for distinguishing active lupus nephritis versus inactive nephritis patients (n = 104) were 44%, 90%, 58% and 84% respectively. These values were similar to the values of other studies included in our analysis. Historically, the early studies, particularly in the 1990s had to make their own lab-based ELISAs (as they were not available commercially). Later studies, from 2003 onwards, frequently used commercially available ELISAs, but no single product has been adopted throughout the lupus-research community. For an immunoassay to be useful in routine clinical practice, clinical laboratories should adopt a single assay procedure. This is the case for measuring autoantibodies against cyclic citrullinated proteins (anti-CCP) as a diagnostic assay for rheumatoid arthritis in which selected commercial ELISAs are approved by the U.S. Food and Drug Administration (FDA).

The inclusion of both ‘lab-made’ and commercial anti-C1q diagnostic ELISAs in our analysis is justified since the assay has evolved with several stringent modifications over the past 30 years. A natural function of C1q is to bind non-specifically to immune complexes. Consequently, using an ELISA method employing whole purified C1q bound to a well of an ELISA plate is susceptible to binding to immune complexes present in the test sera, as well as to antibodies directed against C1q. In some of the earlier studies excluded from our analysis, this problem may have led to a higher number of false positive results. However, it was soon realized that raising the ionic strength of the test buffer to >0.15 M prevented the non-specific binding of immune complexes to solid-phase bound C1q [52], but concerns have been raised that high salt buffers can also prevent anti-C1q autoantibodies binding to C1q [53].Various other potential problems were raised by Siegert, including sera containing double-stranded DNA (dsDNA) that is known to bind to the collagen region of C1q, but increasing the salt concentration of test buffer also alleviates this problem [24]. Another concern is the specificity of the antibodies that bind to C1q. The collagen-like region of C1q bears some homology to type II collagen, which is also a target for autoantibodies in many autoimmune diseases, especially SLE and rheumatoid arthritis. However, a study showed that the autoantibodies directed against type II collagen differed from those that bound to the collagen-like stalks of C1q [54]. We have recently developed a more sophisticated form of ELISA that uses unmodified C1q and post-translationally modified forms of C1q as a target antigen for detecting anti-C1q autoantibodies in SLE sera. This variation may prove to be a more specific and sensitive alternative to current anti-C1q ELISA’s [46,47].

Ten years ago Reveille indicated that the anti-DNA antibody test remained the ‘gold standard’ immunoassay marker for disease activity in SLE, particularly as an indicator of LN [55] with a positive likelihood ratio of 4.41. He also suggested other tests including anti-C1q showed promise in monitoring renal disease in SLE patients. Our study reveals a lack of homogeneity in performing the anti-C1q assay. Despite this, measuring anti-C1q autoantibodies may be a useful diagnostic test for monitoring and detecting evidence of LN in SLE patients, but not as a ‘stand-alone’ assay, but as part of a panel of autoantibodies as has been the recommendation for many years [56]. We would advocate that the anti-C1q immunoassay requires further refinement and development, with greater specificity and sensitivity in gauging LN before a single assay be adopted.

## Figures and Tables

**Table 1:** Summaries of demographic information of the 31studies included in the meta-analysis.

Reference	Country & study date	Patient Number (samples)	% female	Median/Mean Pooled Age (range or Mean ± SD)	Disease duration Years Median (range) or mean ± SD)	Disease criteria & activity Index
**European studies**						
Siegert et al. [30]	Netherlands (1991)	88	91%	37 (15-73)	NR	ACR criteria/SLEDAI
Siegert et al. [31]	Netherlands (1993)	68	96%	38 (14-75)	6 (0.8-24)	ACR criteria/ SLEDAI
Coremans et al. [27]	Netherlands (1995)	33	85%	* 28.9 ± 10.2	* (3.7 ± 3.7)	ACR criteria
				† 34.3 ± 10.1	† (8.9 ± 6.7)	
**Ravelli et al. [57]	Italy (1997)	29	90%	**14.1** (7.5-19.6)	(0.1-14.6)	ACR criteria/ SLEDAI/SLAM
Norsworthy et al. [29]	UK (1999)	195	NR	NR	0.25 – 25	BILAG
Trendelenberg et al. [58]	Switzerland (1999)	48	NR	NR	NR	ACR criteria
Loizou et al. [59]	UK (2000)	56	95%	* **31** (17-61) § **43** (15-74)	20-71	ACR criteria
Moroni et al. [60]	Italy (2001)	48 (61)	92%	* **34** (23-43)	**10.1**	ACR criteria/SLEDAI
				§ **38** (29-49)	**11.3**	
Oelzner et al. [61]	Germany (2003)	79	89%	41.7 ± 13.8	0.25- 30	ACR criteria/SLEDAI
Marto et al. [62]	UK (2005)	151	93%	39 (15-74)	NR	ACR criteria
Sinico et al. [63]	Italy (2005)	61	NR	NR	NR	ACR criteria/ECLAM
Jaekell et al. [64]	Germany (2006)	100	91%	41.7 ± 13.7	NR	ACR criteria/ECLAM
**Kozyro et al. [65]	Switzerland (2006)	12	50%	**15** (10-17)	NR	ACR criteria/SLEDAI
Trendelenberg et al. [66]	Switzerland (2006)	72	NR	NR	NR	ACR criteria
Braun et al. [67]	Germany (2007)	78	88%	37.6 ± 12.3	0.08-33.0	ACR criteria/SLEDAI
Meyer et al. [68]	France (2009)	70	91%	§ **30** (19-58) § **28** (17-48) **35** (20-76)	0.25-36 0.1-14.1 1.1-49.0	ACR criteria/SLEDAI
Smykal-Jankowiak et al. [69]	Poland (2011)	48	100%	* 33.5	5.35	ACR criteria/SLEDAI-2K
**Asian studies**						
Fang et al. [70]	China (2009)	180	84%	* 33 ± 11.34 ¶31.37 ± 11.70	NR	ACR criteria/SLEDAI
Tan et al. [45]	China (2009)	113	NR	NR	NR	ACR criteria/SLEDAI
Cai et al. [71]	China (2010)	73	89%	‡ 31.0 ± 13.9	‡ (2.3 ± 1.6)	ACR criteria/SLEDAI
Mok et al. [72]	China (2010)	245	95%	40.6 ± 12.2	8.7 ± 7.1	ACR criteria/SELENA-SLEDAI
Pradhan et al. [73]	India (2010)	80	NR	NR	NR	ACR criteria/SLEDAI
Katsumata et al. [33]	Japan (2011)	126	98%	**37** (17-77)	NR	ACR criteria/SLEDAI-2K
**Wu et al. [74]	China (2011)	90	87%	9.8 (3-15)	NR	ACR criteria/SLEDAI-2K
Zhang et al. [75]	China (2011)	90	98%	37.08 ± 11.89	4.08	ACR criteria/SLEDAI
				* 34.67 ± 11.21	4.74	
				¶ 39.95 ± 12.12	3.28	
Pradhan et al. [76]	India (2012)	60	92%	29.7 (17-49)	3.6 ± 1.4	ACR criteria/SLEDAI
**North/South American Studies**						
Bernstein et al. [26]	USA (1994)	60	NR	NR	NR	ACR criteria
Haseley et al. [28]	USA (1997)	240	92%	41 ± 9.0	(11 ± 9.0)	ACR criteria
Moura et al. [77]	Brazil (2009)	81	99%	34 ± 11	4 (0.3-32)	ACR criteria/SLEDAI
De Moura et al. [78]	Brazil (2011)	62	85%	* **27.0** ± 5	NR	ACR criteria/SLEDAI
				† **27.5** ± 6.3		
				¶ **28.0** ± 8.0		
**Jesus et al. [79]	Brazil (2012)	67	78%	14.6 ± 3.86	(6.4 ± 3.52)	ACR criteria/SLEDAI-2K

* Active nephritis;

§ Inactive LN;

¶ Non-LN, active SLE with LN;

† active SLE without LN;

§ active SLE without LN;

‡ only LN documented;

**bold text** – median; NR: Not Recorded;

** Pediatric study

**Table 2:** Clinical assessment of nephritis and detection of anti-Clq antibodies in 31 studies.

Reference	Renal Disease Reference standards			Immunoassay used	Cut-off for +ve result
	Biopsy WHO GN types I-VI	Proteinuria(P)/ Creatinine (C)	RBC count/field		
Siegert et al. [30]	13/88 biopsy	P>0.5 g/24 h Increased C	>10	Lab -made	>137 U/ml
Siegert et al. [31]	25/68 biopsy	P>0.5 g/24 h	RBCs in urine	Lab-made	>90 U/ml
Coremans et al. [27]	17/33 biopsy	P>0.5 g/24 h	>5	Lab-made	>90 U/ml
**Ravelli et al. [57]	7/29 biopsy	P>0.5 g/24 h Increased C	>10	Lab-made	>mean 95% OD above 59 HC controls
Norsworthy et al. [29]	37/199 biopsy	P>15 mg/ 24 h	>10	Lab-made	>20 U + 5 SD above controls
Trendelenberg et al. [58]	14/48 biopsy	Abnormal values of P	>20	Lab-made	> 80 U/ml
Loizou et al. [59]	31/56 biopsy	Abnormal P	NR	Lab-made	>20 U + 5 SD above controls
Moroni et al. [60]	biopsy	P>0.5 g/24 h	>5	Lab-made	> 80 U/ml
Oelzner et al. [61]	27/79 biopsy	P ≥ 0.5 g/24 h	NR	IMTEC	≥ 30 U/ml
Marto et al. [62]	77/151 biopsy	NR	NR	Diagenics	>18 U/ml
Sinico et al. [63]	40/61 biopsy	P>2.0 g/24 h	NR	Lab-made	>55 U/ml
Jaekell et al. [64]	Some biopsy	P ≥ 0.5 g/24 h Increased C	NR	Orgentec	>10 U/ml
**Kozyro et al. [65]	12/112 biopsy	P>1 g/L	>20	Bühlmann	>15 U/ml
Trendelenberg et al. [66]	40/72 biopsy	NR	>20	Bühlmann	>40 U/ml
Braun et al. [67]	47/78 biopsy	NR		INOVA	>20 U/ml
Meyer et al. [68]	55/70 biopsy	P>0.5 g/dL/24 h Increased C	Increased RBCs	Bühlmann	>32 U/ml
Smykal-Jankowiak et al. [69]	37/48 Biopsy	P ≥ 0.5g/24 h serum C	Increased RBCs	Bühlmann	>32 U/ml
Fang et al. [70]	Biopsy	P>0.3 g/24 h Increased C	≥ 5	Lab-made	>mean OD + 2 SD above 63 controls
Tan et al. [45]	Biopsy	NR	NR	Lab-made	>mean OD + 2 SD above 100 controls
Cai et al. [71]	Biopsy	P ≥ 0.5 g/24 h	Increased RBCs	IMTEC	>20 U/ml
Mok et al. [72]	NR	NR	NR	Euroimmun	NR
Pradhan et al. [73]	Biopsy	NR	NR	Binding Site	>8 U/ml
Katsumata et al. [33]	20/126 Biopsy	P ≥ 0.5 g/24 h	**NR**	Bühlmann	>40 U/ml
**Wu et al. [74]	28/90 Biopsy	P ≥ 50 mg/Kg/24 h Increased C	NR	Lab-made	>mean OD + 1 SD (40 U/ml) above controls
Zhang et al. [75]	5/49 Biopsy	P ≥ 0.5 g/24 h	Increased RBCs	Euroimmun	≥ 20U/ml
Pradhan et al. [76]	45/60 Biopsy	NR	NR	Autostat II C1q-CIC	≥ 50 μg/ml anti-C1q
Bernstein et al. [26]	8/60 Biopsy	P > 0.5 g/24 h Increased C	NR	Lab-made	>mean OD + 2 SD above 30 inactive SLE controls
Haseley et al. [28]	75/240 Biopsy	P ≥ 0.5 g/24 h Increased C	>10	Lab-made	>mean OD + 5 SD above 30 controls
Moura et al. [77]	No	P > 0.5 g/24 h Increased C	NR	INOVA	>20 U/ml
De Moura et al. [78]	15/62 Biopsy	P ≥ 0.5 g/24 h serum C	NR	Diagenics	≥ 20 U/L
**Jesus et al. [79]	No	P ≥ 0.5g/24 h Increased C	>10	QUATA Lite	>20 U/ml

** Pediatric study

OD: Optical Density; NR: Not Recorded
